# Dietary Flavonoid Intakes Are Associated with Race but Not Income in an Urban Population

**DOI:** 10.3390/nu10111749

**Published:** 2018-11-13

**Authors:** Marie Fanelli Kuczmarski, Rhonda S. Sebastian, Joseph D. Goldman, Theophile Murayi, Lois C. Steinfeldt, Jessica R. Eosso, Alanna J. Moshfegh, Alan B. Zonderman, Michele K. Evans

**Affiliations:** 1 Department of Behavioral Health and Nutrition, University of Delaware, Newark, DE 19716, USA; mfk@udel.edu (M.F.K.); jeosso@udel.edu (J.R.E.); 2 Food Surveys Research Group, Beltsville Human Nutrition Research Center, Agricultural Research Service, USDA, Beltsville, MD 20705, USA; Joe.Goldman@usda.gov (J.D.G.); Theo.Murayi@usda.gov (T.M.); Lois.Steinfeldt@usda.gov (L.C.S.); Alanna.Moshfegh@usda.gov (A.J.M.); 3 Laboratory of Epidemiology and Population Sciences, National Institute on Aging, National Institute of Health, Baltimore, MD 21224, USA; zondermana@mail.nih.gov (A.B.Z.); evansm@grc.nia.nih.gov (M.K.E.)

**Keywords:** flavonoids, polyphenols, African Americans, HANDLS, What We Eat in America, NHANES, diet, disparities

## Abstract

Flavonoids are polyphenolic phytochemicals with health-promoting properties, yet knowledge about their intake in at-risk populations is limited. This study sought to estimate intakes of total flavonoids and six flavonoid classes in the Healthy Aging in Neighborhoods of Diversity across the Life Span (HANDLS) study; determine if differences in intakes exist by race (African American (AA) and White (W)) and income (< or >125% Federal poverty guidelines); and compare intakes to those of a nationally representative population with similar demographic and socioeconomic characteristics. Data transformation normalized the flavonoid intake distributions prior to conducting statistical tests. With the exception of the flavanone class, flavonoid intakes of AAs were significantly lower than those of W (*p* < 0.01), regardless of other potential mediating factors including sex, age, and income. Total flavonoid intakes in HANDLS did not differ from intakes in the nationally representative study, but anthocyanidin and flavone intakes were lower, and race specific differences were found for several flavonoid classes. These findings imply that benefits attributable to flavonoid consumption may not be experienced equally by AAs and Whites, nor in vulnerable populations such as that represented by HANDLS relative to the U.S. population, and may play a role in observed health disparities.

## 1. Introduction

African Americans (AA) are at greater risk for developing chronic diseases compared to White (W) Americans [1]. Socioeconomic status and lifestyle factors, especially diet, contribute to these health disparities. In fact, diet is considered one of the strongest modulators of chronic inflammation [2,3], a condition associated with the development of obesity, cardiovascular disease, cancer and diabetes [4,5,6]. It has been estimated that half of all cardiovascular events could be prevented by improving the diet [7].

Diets are composed of foods and beverages which contain not only macro- and micronutrients but also phytochemicals. Some of these compounds, such as carotenoids and polyphenols, may promote health and prevent disease [8,9]. Flavonoids are the most abundant polyphenols in the diet, accounting for approximately two-thirds of intake [10]. Based on their chemical structure, they may be categorized into six classes: anthocyanidins, flavan-3-ols, flavanones, flavones, flavonols, and isoflavones [11].

In vitro, the health-promoting actions of flavonoids are well documented. They have been shown to exhibit anti-inflammatory and antioxidant properties [12,13,14]. It is apparent that these actions have implications for a variety of chronic diseases. Indeed, extensive research has found inverse associations between flavonoid intake and chronic diseases including cardiovascular disease [7,15,16,17,18], cancer [19,20,21,22,23], diabetes [24,25,26,27], and obesity [28,29,30].

Despite their purported health benefits, knowledge about the flavonoid intake of at-risk populations is limited. In most large-scale investigations of flavonoid-health associations, the study sample was drawn from fairly homogenous populations. For example, in the Nurse’s Health Study I and II [31], and the Health Professional’s Follow-up Study [32], 97% of study participants were white and all were college educated [33,34]. One notable exception is the REGARDS (Reasons for Geographic and Racial Differences in Stroke) study, a national prospective cohort of community dwelling adults, which included a large proportion (42%) of black Americans [35,36]. Inverse associations between anthocyanidin intake and incident coronary heart disease [35] and flavanone intake and incident ischemic stroke in both whites and blacks [36] were reported. However, the design of the REGARDS study did not consider socioeconomic status when selecting the sample, nor were investigations of flavonoid intake-disease associations stratified by income. In addition, since REGARDS was focused on associations with stroke incidence, much of the sample was selected in the “Stroke Belt”, which encompasses the southern United States (U.S.) and includes rural, suburban, and urban communities [35,36,37].

The Healthy Aging in Neighborhoods of Diversity across the Life Span (HANDLS) study was designed specifically to measure the associations between race and socioeconomic status, both independently and synergistically, and health disparities [38]. Previously published research indicates the prevalence of risky health behaviors, including a nutrient-poor diet, is high among participants in HANDLS. For instance, Kuczmarski et al. found that most HANDLS participants consume a pro-inflammatory Westernized diet [39]. The Westernized diet is characterized by high intakes of saturated fat, proteins (derived from processed meats), and sugars and low intakes of dietary fiber [40]. Though it is assumed to be far less than optimal, flavonoid intakes, and thus the potential health benefits they could confer, are not known for this population. Further, whether these intakes differ by race and income is similarly not determined.

The objectives of this study were: (1) estimate flavonoid intakes of a large, racially and socioeconomically diverse urban population; (2) characterize major food and/or beverage contributors to intakes of total flavonoids and six flavonoid classes by race and income; (3) identify differences in intake between AA and W; and (4) identify differences in intake between higher and lower-income groups. Finally, because there is some evidence that AA have lower flavonoid intakes than W [41], flavonoid intake estimates in the HANDLS study will be compared, for all and by race, to those of a nationally representative sample that is similar with regards to race and income. The purpose of this contrast is to ascertain if intakes observed in HANDLS were unique to this at-risk population or simply a reflection of intakes observed for the U.S. population as a whole.

## 2. Materials and Methods

### 2.1. HANDLS Study Sample

The HANDLS study is a prospective longitudinal (20 years) epidemiologic investigation of AA and W urban adults. The baseline sample, which was analyzed in this study, was initiated in 2004 and completed in 2009. Participants were drawn from 13 pre-determined neighborhoods, comprising an area probability sample of Baltimore City. The design was a 4-way factorial cross of age (seven five-year age bands between 30–64 years), sex (men and women), race (self-reported; non-Hispanic AA and non-Hispanic W), and income (self-reported household income <125% and >125% of the 2004 Health and Human Services poverty guidelines; hereafter termed “poverty”; [42]). Inclusion criteria for the study were ability to: (1) give informed consent, (2) provide valid photo identification, and (3) perform at least 5 of the following evaluations: medical history, physical performance, cognitive testing, dietary recall, audio questionnaire, body composition, and carotid Doppler or pulse-wave velocity assessment. Exclusion criteria were pregnancy, AIDS (Acquired Immune Deficiency Syndrome) diagnosis, and cancer treatment within the last 6 months [38,43].

Phase 1 of the baseline visit included household screening and recruitment. If eligible persons were identified, a general household questionnaire and one 24-h dietary recall were administered. All information on the household questionnaire was self-reported, and included educational experience, occupational history, family income, health status (Short Form 12-item survey [44]), and other data related to psychosocial factors and neighborhood characteristics [43]. Phase 2 consisted of in-depth examinations in the study’s Mobile Research Vehicles [38,43]. At that visit, literacy, psychosocial, anthropometric, and physiologic measurements were collected as well as a fasting blood draw and a second 24-h dietary recall. Questions related to health behaviors such as tobacco cigarette smoking, stress, and coping were collected by ACASI (audio computer-assisted self-interview software). Literacy was assessed using the reading subtest of the Wide Range Achievement Test-3rd Edition (WRAT-3) [45].

The baseline sample consisted of 3720 persons, of whom 91.9% completed at least one dietary recall, and 59.2% completed both recalls. Individuals who declined to participate in Phase 2 and thus did not complete the second dietary recall were shown to differ significantly in sex, age, and race from individuals who completed the examination. Older participants (aged 48–64 years), women, and W were more likely to complete Phase 2 [38]. To avoid this inherent nonresponse bias, only one day of dietary data from all participants was analyzed.

The study protocol was approved by the human investigation review boards at both MedStar Health Research Institute and the University of Delaware. All HANDLS participants provided written informed consent and were compensated monetarily.

### 2.2. Food Intake

Dietary intakes in this study were estimated via one 24-h recall, which was collected by trained interviewers using the U.S. Department of Agriculture (USDA) Automated Multiple-Pass Method (AMPM) [46,47]. Measurement aids, including measuring cups, spoons, ruler, and an illustrated food model booklet, assisted participants in estimating accurate quantities of foods and beverages consumed. Recalls were coded using the Food and Nutrient Database for Dietary Studies (FNDDS) 3.0. Though its primary purpose is to process dietary data in What We Eat in America (WWEIA), National Health and Nutrition Examination Survey (NHANES), the FNDDS may be applied in other studies such as HANDLS to code foods and amounts eaten and calculate nutrient and food component intakes [48]. Using the WWEIA Food Categories as a foundation, each food/beverage in FNDDS was classified into one of 71 mutually exclusive groups [49].

### 2.3. Flavonoid Intake

Though the FNDDS includes over 60 nutrients and food components, it does not include flavonoids. The Flavonoid Values for USDA Survey Foods and Beverages 2007–2010 (Flavonoid Database) fills this gap [50]. The development of this special use database is described elsewhere [41]. It provides flavonoid values for all foods/beverages in FNDDS 5.0. The USDA food codes serve as the link between FNDDS and the Flavonoid Database. For this study, dietary intakes of total flavonoids and six flavonoid classes (anthocyanidins, flavan-3-ols, flavanones, flavones, flavonols, and isoflavones) were estimated. Any contribution to flavonoid intake from dietary supplements was not assessed and thus not included.

Since recalls from the HANDLS study were coded using FNDDS 3.0 and the Flavonoid Database corresponds to FNDDS 5.0, there were some foods reported that were assigned food codes that were not available in FNDDS 5.0. Food codes lacking a flavonoid profile in the Flavonoid Database (4 foods; 3 pizzas and 1 cereal) were assigned the flavonoid profile of the food code most similar in content of flavonoid-containing ingredients. Accordingly, all food/beverage items reported by HANDLS participants were included in the calculation of flavonoid intakes.

### 2.4. Statistical Analysis

Data preparation, transformation and analysis were performed using SAS^®^/BASE and SAS^®^/STAT, release 9.4 (2012; SAS Institute Inc., Cary, NC, USA). SAS-callable SUDAAN^®^, release 11.0 (2012; RTI International, Research Triangle Park, NC, USA) was used to account for the HANDLS sample design in statistical testing. Sample weights were applied to permit calculation of estimates representative of the Baltimore City AA and W adult population.

To identify important contributors to dietary intake of flavonoids among the HANDLS population, the percentage contribution of each of the 71 food/beverage categories to intakes of total flavonoids and each flavonoid class was calculated. Food groups that contributed 10 percent or more to total flavonoid or any flavonoid class intake were identified for the HANDLS population overall, by race (AA, W), and by income category (<125% and >125% poverty) [42].

Prior to conducting between group comparisons, the distributions of total flavonoid and flavonoid class intakes were assessed. All were extremely positively skewed, corroborated by significant (*p* < 0.05) Kolmogov-Smirnov (K-S) test statistics. Data transformation was required to meet the normality assumption underlying parametric testing. The Markov Chain Monte Carlo hierarchical predictive modeling transformation successfully normalized the flavonoid intake distributions [51]. Following application of this method, all flavonoid intake distributions yielded non-significant (*p* > 0.05) K-S test statistics, with mean to median ratios very near 1.00 (ranging from 0.95 to 1.04).

Linear regression produced adjusted estimates of total flavonoid and flavonoid class intake by race and income individually and by race and income within the remaining factors that defined the study sample (sex; age; race or income). Adjustment variables included in the analyses as appropriate were energy, sex, income, and age. Though flavonoid intake by race and income were tested by age category (30–49 years and 50–64 years), age was entered as a centered, continuous variable when applied in adjustment. Differences in flavonoid intake estimates by race and income were determined using *t*-tests, and *p* values reflect a comparison of estimates derived from the transformed data.

To evaluate whether flavonoid intakes in HANDLS differed from those of a nationally representative sample with similar socioeconomic characteristics, a subsample of individuals from WWEIA, NHANES 2007–2010 was selected. All individuals who met the following criteria were included: (1) 30–64 years of age, (2) non-Hispanic white or non-Hispanic black, and (3) household income ≤$75,000 per year. Pregnant and lactating females were excluded, yielding a final sample of 2598 adults. Like HANDLS, the WWEIA, NHANES flavonoid data distributions were severely positively skewed, and thus were transformed in the same manner as the HANDLS flavonoid data [51]. Adjusted flavonoid intake estimates were calculated for all adults in HANDLS and the WWEIA, NHANES subsample and for AA and W (HANDLS) and blacks and whites (WWEIA, NHANES) separately. *p* values for differences between surveys are based on results of *t*-tests on the transformed data. As in analyses of HANDLS exclusively, the sample design was accounted for in analysis of WWEIA, NHANES, and the sample weights were applied in order to produce nationally representative flavonoid intake estimates.

In all analyses, results with a probability of occurrence of *p* < 0.01 were considered statistically significant. This somewhat conservative significance level was selected to address the potential for drawing false inferences due to the multiple comparisons conducted while simultaneously acknowledging the highly correlated nature of the flavonoid intake data.

## 3. Results

### 3.1. Participant Characteristics

A total of 3418 adults (2029 AA and 1389 W) provided one complete day of dietary intake data. Demographic and lifestyle characteristics are shown in Table 1. More than half of the sample (59.4%) was AA. Slightly less than half (41.6%) had incomes less than 125% of poverty (for perspective, 125% of poverty equates to an annual income of $18,850 for a 4-person household in 2004). Approximately one-third of HANDLS participants had not completed high school or its equivalent, and about the same percentage self-assessed their health status as excellent or very good. Only a slight majority of participants reported that they were employed last month. 

As noted earlier, several measurements, including WRAT score, BMI, and smoking status were collected during Phase 2 of the HANDLS study. Since some individuals declined to continue participation after the Phase 1 interview, these data were never collected for a proportion of the study sample. The majority of the “not available” responses for these variables reflect this nonresponse (Table 1).

### 3.2. Flavonoid Intakes and Dietary Sources

The mean (±SE) total flavonoid intake of the HANDLS study population was 225.36 ± 32.57 mg/day (Table 2). Some flavonoid classes exhibited a large percentage of zero intakes, namely, anthocyanidins (54.5%), and flavanones (46.0%), and isoflavones (69.7%).

The food groups that contributed 10% or more of total intake of flavonoids and flavonoid classes are shown in Table 3. In general, identified food groups were fairly consistent across race and income subgroups. For the HANDLS population as a whole and by race and income, tea was the only food group that contributed ≥10% to daily intake of total flavonoids and flavan-3-ols, and it accounted for the largest proportion of flavonol intake of any food group. Tea was also a notable source of flavones. Likewise, orange juice contributed the largest proportion to flavanone intake and mixed dishes to flavonol intake regardless of race and income. The food groups that contributed 10% or more to intake of the anthocyanidin and isoflavones classes were similar across race and income, though the rank order of a particular food group varied, and some foods were listed for some race or income groups and not for others. For example, berries contributed ≥10% of total anthocyanidin intake for W and those in the >125% income category, but not for AA or those in the <125% category.

### 3.3. Flavonoid Intakes by Race and Income 

As shown in Table 4, mean intakes of total flavonoids, and all six flavonoid classes were different by race (*p* < 0.01). When examining racial differences overall and by sex, income, and age, intake of total flavonoids, anthocyanidins, flavan-3-ols, flavones, flavonols, and isoflavones were lower among AA as compared to W. Among all individuals, total flavonoid intake was approximately 47% lower among AA than W, and intakes of the five classes listed were 27% (flavonol) to 62% (anthocyanidins) lower. On the other hand, estimates of flavanone intake were higher among AA versus that of W, regardless of sex, income, or age group.

In stark contrast, income was not significantly associated with flavonoid intake for all adults, nor within sex, race, and age (Table 5).

### 3.4. Comparison of Flavonoid Intakes in HANDLS and WWEIA, NHANES

Although total flavonoid intake estimates of the HANDLS population did not differ relative to those of a comparable (i.e., with similar demographic characteristics) population from WWEIA, NHANES, 2007–2010, anthocyanidin and flavone intakes were significantly lower in HANDLS (*p* < 0.01; Table 6). Intake of the other flavonoid classes overall (both races and income categories together) did not differ between HANDLS and WWEIA, NHANES.

Analyses by race yielded dissimilar findings. Anthocyanidin intakes among both AA and W were lower in HANDLS as compared to WWEIA, NHANES. However, the remainder of the flavonoid intake differences between populations varied by race. Among AA/blacks only, flavone and isoflavones intakes were lower in HANDLS, whereas among W, intakes of flavan-3-ols, isoflavones, and total flavonoids were higher relative to estimates derived from WWEIA, NHANES (Table 6).

## 4. Discussion

In this study, factors that are typically predictive of nutritional status and thus likelihood of nutrition-related disease were investigated in relation to their associations with flavonoid intake. These findings provide the first evidence that race, regardless of other potential mediating variables including sex, age, and income, was associated with differences in flavonoid intake in a large, at-risk population. With the sole exception of the flavanone class, intakes of flavonoids among AA were significantly lower than those of W. Conversely, income was not related to flavonoid intake.

It is difficult to incorporate these results with existing literature because flavonoid intake has not been investigated by race and income within other relevant characteristics. However, a few studies have analyzed flavonoid intake by race and income individually, and thus can be compared to our overall findings. In an investigation of flavonoid-incident ischemic stroke associations in REGARDS, Goetz and colleagues reported that AA had higher flavanone intakes as compared to W, while intake of total flavonoids and the other flavonoid classes were higher among W, though it is not clear if statistical testing was conducted [36]. Analyses of WWEIA, NHANES data have also found racial differences in flavonoid intake. Sebastian et al. reported that non-Hispanic blacks had lower total flavonoid intake as compared to non-Hispanic whites or Hispanics [41]. Differences in flavonoid class intake by race in earlier NHANES cycles have also been reported [52], though which groups differed significantly in intake from others were not elucidated.

The relationship between flavonoid intake and income is less consistent. The majority of households included in the HANDLS study had incomes below $75,000 [53], which may have contributed to the observed lack of association with flavonoid intake. Financial resources could have been a limiting factor to achieving a diet replete in fruits and vegetables for all persons in the HANDLS population, and not just those in the lower-income category. This assertion is supported by earlier research that reported mean total HEI-2010 scores in baseline HANDLS participants were approximately 10 points lower than those of the U.S. population [54]. It is well documented that lower quality diets cost less, and the foods they include are commonly selected by lower-income individuals [55]. Nevertheless, these findings of no association concur with results from some studies, but not others. Using transformed flavonoid data from WWEIA, NHANES, which has a greater range of incomes than HANDLS, previously reported research found no differences in total flavonoid intake by income [41]. However, other work analyzing earlier NHANES data cycles did report intake differences by income [52,56]. These later studies either did not transform the flavonoid data [56] or used log transformation [52], which proved inadequate to normalize the flavonoid distribution in the present study. In addition, those studies were not able to fully estimate flavonoid intake from all foods and beverages due to missing flavonoid composition data, which might account for the inconsistency with the current findings [52,56].

There may be many factors influencing the lower consumption of flavonoids by AA. Food selection decisions are complex, dependent on interactions between behavior and biology, and modulated by environment and community structure [57]. Disparate intake of flavonoid classes by race could be a reflection of cultural food habits [11,58]. Previous research of HANDLS data has revealed food selections varied by race [39,54], which contributed to observed differences in dietary patterns with different 10-year atherosclerotic cardiovascular disease risk [39]. When compared to AA, W had higher mean Healthy Eating Index (HEI)-2010 scores for the total vegetables and whole fruits components [59], which would be consistent with W’s observed higher intakes of flavones and flavonols (both found primarily in vegetables) and anthocyanidins (found primarily in fruits, particularly berries and grapes). Similarly, it is clear that AA had higher intakes of the foods that are the primary contributors of flavanones in the American diet. Orange juice was the highest contributor of flavanone intake regardless of race. Post hoc analyses of HANDLS revealed that although the adjusted percentage of adults reporting orange juice did not differ by race (16 percent vs. 10 percent for AA and W, respectively; *p* = 0.011), the adjusted mean amount consumed by AA (60 gm) was more than double that consumed by W (29 gm; *p* < 0.01). Orange juice contains ~19 mg of flavanones per 100 gm [50]. Based upon the intake estimates presented in this study, it can be concluded that orange juice intake alone accounted for approximately 80 percent of the difference in flavanone intake observed between AA and W.

Food availability is another factor that must be considered when disparities in intake are found. The concept of “food deserts”, defined as geographic areas with low availability or high prices of healthy foods, has been a popular one in recent years [60]. It provides a rationale for why individuals who reside in areas with low healthful food availability may consume nutrient- and polyphenol-poor diets. However, its influence on dietary choice may be overstated [60]. Instead, it may be that effort to obtain healthful foods [61] (in addition to well-established economic considerations previously mentioned [57]) is a more important determinant of fruit and vegetable and thus flavonoid intake than food availability [57,61], because travel outside one’s immediate neighborhood to acquire food is common [57,60]. This food shopping behavior is evident and impactful. Previous analyses of HANDLS revealed that, contrary to expectations, individuals who lived in neighborhoods with low healthy food availability had better diet quality than did their counterparts who resided in neighborhoods with medium and high healthy food availability [61], suggesting that those in the former group traveled to obtain food. In another study also among an urban population, shopping at convenience and neighborhood stores, which are prevalent in the immediate environment, was positively associated with intake of added sugars and discretionary fat. Conversely, shopping at the types of stores that are typically not present in lower-income urban areas, such as specialty grocery stores and full-service supermarkets, was positively associated with fruit and vegetable intake [62]. Furthermore, urban W adults appear to be more likely to travel to shop as compared to AA adults [61]. The fact that W engage in this behavior to a greater extent than AA and thus potentially have more healthful choices available could explain their higher intake of total flavonoids and most flavonoid classes analyzed. This behavior is an area that deserves further study.

Other attributes of flavonoid-rich foods could potentially be important with respect to the differences observed in flavonoid intake between the exclusively urban HANDLS population and the comparable NHANES population. Anthocyanidin intakes in HANDLS were lower overall and by race relative to intakes in WWEIA, NHANES, due in part to the greater percentage in HANDLS with no intake of anthocyanidins at all (54.4 ± 1.2% vs 39.6 ± 1.9% in NHANES; unpublished NHANES data). Berries and grapes are primary sources of anthocyanidins in HANDLS and NHANES [41]. Both are relatively expensive and perishable fruits, which are qualities that may discourage their purchase in a population who, besides having limited financial resources, may have to travel some distance to obtain them.

When flavonoid intake estimates in HANDLS and NHANES were compared within race, differences emerged. Among AA/blacks, for those flavonoid classes that differed between the surveys, intakes were consistently lower in the HANDLS population. In contrast, among W, intakes of anthocyanidins were lower in HANDLS but intake of total flavonoids and the flavan-3-ol and isoflavones classes were higher. These types of results exemplify the need to look at subpopulations individually, because disparate associations could lead to a conclusion of no relation for the population as a whole.

Overall, it may be that the unique characteristics of HANDLS play an important role in accounting for differences in flavonoid intake. The HANDLS sample, by design, targeted an at-risk population. As such, factors typically associated with health and health disparities beyond race and income are more pronounced in HANDLS as compared to the U.S. population as a whole. For instance, in 2005, among adults 25–64 years in the U.S., 12% did not have a high school degree or its equivalent [63]. In the HANDLS sample, fully one-third fit this criterion. Education has been shown to be positively associated with intake of multiple Healthy Eating Index fruit and vegetable components–total vegetables, greens and beans, total fruit, and whole fruit [54], all of which are positively associated with flavonoid intake [41]. In addition, as employment status is highly correlated with education, it is not surprising that the percentage employed in the HANDLS sample (~56%) appears lower than 2005 national estimates (75.5%) [64]. Unemployment status is another characteristic that has been shown to be negatively associated with fruit and vegetable consumption [65]. It appears that lower intake of flavonoids is yet another marker of compromised dietary intake that places the population represented by HANDLS at greater nutritional risk [39,66,67].

As with any research there are strengths and limitations. One major strength is that the results were based on analyses of data that was transformed to meet the assumptions of the statistical tests. Another strength is the relatively large sample of African Americans, a feature that is atypical of most studies investigating flavonoid intake. The flavonoid composition data and dietary data analyzed are other assets of this study. Use of the Flavonoid Database for USDA Food Codes permitted comprehensive estimation of flavonoid intake with minimal imputation to address missing flavonoid profiles for foods. The dietary collection method used in both HANDLS and WWEIA, NHANES, the USDA AMPM, has been shown to reduce bias in the collection of energy intakes, and to provide accurate estimates of sodium intake [46,68]. Nevertheless, inherent errors are associated with the 24-h recall [69]. Furthermore, there are a plethora of other issues to consider with the use of self-reported dietary data that extends beyond the dietary assessment method. Social desirability can affect reporting of behaviors such as food and beverage intake. However, there seems to be no consistent pattern of either under- or over-reporting of healthful foods like fruits and vegetables [70], so flavonoid estimation is likely minimally affected. Lastly, this analysis of one-day 24-h intakes per sample person does not address the long-term consumption of flavonoids. Rather, it provides a picture of daily mean intake of flavonoids for the HANDLS population on any given day of the year.

## 5. Conclusions

In this understudied urban population, flavonoid intake estimates differed by race, but not by income. These findings imply that the health-promoting benefits attributable to flavonoid consumption may not be experienced equally by AA and W adults. Moreover, flavonoid intake in HANDLS differed from that of a comparable nationally representative sample, suggesting variables other than race and income may be relevant, and could include financial and geographical access to food, as well as behavioral, biological, and environmental factors. Ascertaining the relationship between these factors and flavonoid intake was beyond the scope of this study, but is an area that deserves further investigation.

The lower flavonoid intakes of AA relative to W could potentially play a role in observed racial health disparities, especially with regards to cardiovascular and metabolic diseases that most significantly affect health outcomes and longevity. Given these differences, further research is warranted to ascertain whether previously established favorable associations between flavonoid intake and markers and development of chronic disease hold true for both racial groups in this high-risk population.

## Figures and Tables

**Table 1:** Characteristics of HANDLS participants, 30–64 years, baseline sample (*n* = 3418).

Characteristic	Percent
*Demographic:*	
Male ^1^	45.3
Age 30–49 years ^1^	55.2
African American ^1^	59.4
Income <125% Poverty ^1,2^	41.6
Education level ^3^	
<High school diploma/GED	33.4
High school diploma/GED	33.5
Post-secondary education	31.1
Not reported	2.0
WRAT score ^4^	
≤8th	28.3
9th–12th	19.4
>12th	26.1
Not available ^5^	26.2
*Lifestyle:*	
Body Mass Index (BMI)	
≤25 kg/m^2^	22.4
25.1–29.9 kg/m^2^	21.7
≥30 kg/m^2^	33.2
Not available ^5^	22.7
Cigarette smoking status	
Currently smoking	33.9
Not currently smoking	35.9
Not available ^5^	30.2
Employed last month ^1,3^	
Yes	55.9
No	42.2
Not reported	1.8
Health status ^3,6^	
Excellent/very good	33.0
Good	40.3
Fair/poor	26.7
Not reported	<0.1

^1^ For dichotomous variables with no missing data, the percentage of individuals classified in one of the two possible alternatives for that characteristic is presented on a single line (all such values). ^2^ Health and Human Services 2004 poverty guidelines, income expressed as a percentage of poverty [42]. ^3^ For the specified variable, percentages do not add up to 100 due to rounding. ^4^ WRAT (Wide Range Achievement Test) score is a measure of word reading, sentence comprehension, spelling, and math computation skills expressed as grade equivalents [45]. ^5^ WRAT score, BMI, and smoking status information were collected in the mobile examination vehicle on a date following the interview conducted in the respondent’s home. The majority of the “not available” responses for these variables reflect individuals who declined to participate after the household interview. ^6^ Determined by Short Form 12-item survey [44].

**Table 2:** Dietary intake ^1^ (mg) of flavonoids, HANDLS, 2004–2009, 1 day.

Flavonoid Class	Mean (SE)	Percentage with Zero Intake (SE)	Percentile				
			10	25	50	75	90
Anthocyanidins	7.03 (0.36)	54.4 (1.2)	0.00	0.00	0.00	2.30	14.95
Flavan-3-ols	184.62 (32.04)	15.1 (0.8)	0.00	0.55	7.27	69.41	602.99
Flavanones	14.47 (2.06)	46.0 (1.4)	0.00	0.00	0.11	5.39	55.78
Flavones	0.63 (0.04)	19.5 (1.3)	0.00	0.02	0.22	0.66	1.61
Flavonols	17.65 (1.39)	2.3 (0.3)	1.33	4.03	9.98	21.85	41.39
Isoflavones ^2^	0.96 (0.15)	69.7 (1.7)	0.00	0.00	0.00	0.01	0.66
Total flavonoids ^3^	225.36 (32.57)	2.0 (0.4)	3.55	11.42	36.89	196.07	677.41

Abbreviations: HANDLS, Healthy Aging in Neighborhoods of Diversity across the Life Span ^1^ To permit extrapolation to the population of interest, sample weights were applied to all estimates and the sample design was accounted for in the calculation of standard errors. ^2^ Excludes some isoflavones contributed by functional ingredients added in small amounts to foods and beverages, as outlined in the documentation for the Flavonoid Database for USDA Survey Foods and Beverages [50]. ^3^ Sum of dietary flavonoids in the six classes listed.

**Table 3:** Food/beverage groups ^1^ contributing ≥10% of total intake of flavonoids and flavonoid classes, for all, by race and by income ^2^ category, HANDLS, 1 day.

Flavonoid Class	Rank	All	Race		Income Category ^2^	
			AA	White	<125%	>125%
Total flavonoids	1	Tea (82)	Tea (77)	Tea (86)	Tea (85)	Tea (81)
Anthocyanidins	1	Berries (23)	Wine (25)	Berries (33)	Non-citrus juice, 100% (24)	Berries (24)
	2	Wine (22)	Grapes (15)	Wine (20)	Wine (14)	Wine (23)
	3	Grapes (13)	Non-citrus juice, 100% (15)	Grapes (11)		Grapes (13)
	4	Non-citrus juice, 100% (10)				
Flavan-3-ols	1	Tea (96)	Tea (95)	Tea (96)	Tea (97)	Tea (96)
Flavanones	1	Orange juice (66)	Orange juice (69)	Orange juice (59)	Orange juice (64)	Orange juice (67)
	2	Oranges (14)	Oranges (13)	Oranges (16)	Oranges (13)	Oranges (14)
Flavones	1	Mixed dishes ^3^ (21)	Mixed dishes ^3^ (25)	Mixed dishes ^3^ (17)	Mixed dishes ^3^ (25)	Mixed dishes ^3^ (20)
	2	Sweet peppers (15)	Sweet peppers (16)	Tea (15)	Tea (18)	Sweet peppers (15)
	3	Tea (14)	Tea (12)	Sweet peppers (13)	Sweet peppers (11)	Tea (13)
Flavonols	1	Tea (39)	Tea (32)	Tea (49)	Tea (38)	Tea (40)
	2	Onions (10)	Onions (13)		Dark green vegetables ^4^ (10)	Onions (11)
	3		Mixed dishes ^3^ (10)			
Isoflavones	1	Soy products ^5^ (28)	Soy products ^5^ (44)	Milk substitutes (30)	Soups (25)	Soy products ^5^ (29)
	2	Protein powders (23)	Soups (16)	Protein powders (29)	Soy products ^5^ (24)	Protein powders (25)
	3	Milk substitutes (21)	Protein powders (15)	Soy products ^5^ (17)	Doughnuts ^6^ (18)	Milk substitutes (22)
	4	Soups (10)	Doughnuts ^6^ (10)		Milk substitutes (14)	
	5				Protein powders (10)	

Abbreviations: HANDLS, Healthy Aging in Neighborhoods of Diversity across the Life Span; AA, African American ^1^ To permit extrapolation to the population of interest, sample weights were applied to all estimates. ^2^ Health and Human Services 2004 poverty guidelines, income expressed as a percentage of poverty [42]. ^3^ Corresponds to the What We Eat in America Food Categories [49] listed under “Mixed dishes”, except for Soups. ^4^ Excludes romaine, which is categorized as lettuce [49]. ^5^ Includes processed soy-based foods such as soybean curd (tofu), meat substitutes, and mixed dishes that contain meat substitutes. ^6^ Includes sweet rolls and pastries.

**Table 4:** Mean flavonoid intakes ^1,2^ (mg) by race within selected characteristics, HANDLS, 1 day.

Race by Selected Characteristics	*n*	Flavonoid Class						Total Flavonoids
		Anthocyanidins	Flavan-3-ols	Flavanones	Flavones	Flavonols	Isoflavones	
		*-----------------------------------------------------------milligrams (SE)-----------------------------------------------------------*						
**All adults**								
African American	2.029	4.48 * (0.28)	133.53 * (11.89)	16.99 * (2.37)	0.51 * (0.04)	15.58 * (0.80)	0.62 * (0.11)	171.72 * (11.03)
White	1389	11.77 (1.62)	279.39 (72.34)	9.78 (1.17)	0.86 (0.11)	21.48 (2.13)	1.58 (0.58)	324.85 (71.43)
**Sex**								
*Males*								
African American	922	4.10 * (0.82)	128.76 * (22.95)	20.24 * (3.36)	0.57 * (0.10)	17.49 * (0.99)	0.88 * (0.19)	172.03 * (19.71)
White	628	10.24 (2.72)	240.93 (56.73)	12.34 (1.18)	0.83 (0.11)	21.26 (1.79)	1.15(0.45)	286.74 (55.61)
*Females*								
African American	1107	4.84 * (0.48)	138.34 * (6.81)	14.10 * (1.87)	0.46 * (0.03)	13.91 * (0.61)	0.40 * (0.08)	172.05 * (7.61)
White	761	13.17 (0.92)	315.19 (87.04)	7.55 (2.00)	0.90 (0.12)	21.80 (2.46)	1.99 (0.76)	360.59 (86.25)
**Income ^3^**								
*<125%*								
African American	982	3.56 * (0.65)	117.32 * (12.94)	11.93 * (2.41)	0.38 * (0.02)	14.94 * (0.75)	0.39 * (0.11)	148.52 * (11.09)
White	439	4.33 (1.54)	378.23 (103.41)	8.20 (2.05)	0.67 (0.10)	24.36 (2.25)	0.84 (0.40)	416.62 (103.63)
*>125%*								
African American	1047	4.61 * (0.30)	138.89 * (12.38)	18.23 * (2.48)	0.54 * (0.04)	15.78 * (1.01)	0.67 * (0.13)	178.72 * (11.94)
White	950	13.02 (1.60)	264.83 (65.96)	10.44 (1.22)	0.90 (0.11)	21.08 (2.13)	1.71 (0.62)	311.99 (64.89)
**Age, years**								
*30–49*								
African American	1131	4.73 * (0.72)	138.61 * (14.39)	17.15 * (2.54)	0.52 * (0.05)	16.09 * (0.86)	0.78 * (0.18)	177.88 * (13.71)
White	756	10.33 (3.16)	298.60 (76.18)	7.56 (0.54)	0.93 (0.14)	22.10 (2.34)	1.12 (0.23)	340.63 (75.12)
*50–64*								
African American	898	4.11 * (0.89)	122.60 * (11.95)	16.94 * (3.73)	0.51 * (0.03)	14.69 * (1.01)	0.37 * (0.04)	159.21 * (11.50)
White	633	13.96 (1.43)	255.78 (62.98)	12.84 (2.71)	0.75 (0.08)	20.76 (1.72)	2.31 (1.12)	306.40 (62.47)

Abbreviations: HANDLS, Healthy Aging in Neighborhoods of Diversity across the Life Span * Within characteristic and flavonoid class, intake estimate is significantly different from that of whites (*p* < 0.01). ^1^ To permit extrapolation to the population of interest, sample weights were applied to all estimates and the sample design was accounted for in the calculation of standard errors. ^2^ Estimates adjusted for energy, sex (as appropriate), income (as appropriate), and age. Although untransformed estimates are presented, significance results are based on transformed estimates [51]. ^3^ Health and Human Services 2004 poverty guidelines, income expressed as a percentage of poverty [42].

**Table 5:** Mean flavonoid intakes ^1,2^ (mg) by income ^3^ within selected characteristics, HANDLS, 1 day.

Income by Selected Characteristics	*n*	Flavonoid Class						Total Flavonoids
		Anthocyanidins	Flavan-3-ols	Flavanones	Flavones	Flavonols	Isoflavones	
		*-----------------------------------------------------------milligrams (SE)-----------------------------------------------------------*						
**All adults**								
<125%	1421	4.52 (1.37)	192.88 (39.86)	10.64 (1.90)	0.50 (0.05)	18.13 (0.78)	0.62 (0.19)	227.29 (38.88)
>125%	1997	7.63 (0.45)	182.66 (14.36)	15.37 (1.71)	0.67 (0.05)	17.54 (0.73)	1.04 (0.25)	224.90 (14.07)
**Sex**								
*Males*								
<125%	601	5.42 (1.99)	199.79 (55.55)	12.44 (1.33)	0.54 (0.03)	18.98 (1.59)	0.82 (0.27)	237.99 (54.51)
>125%	949	6.52 (0.71)	164.04 (11.73)	18.29 (2.66)	0.68 (0.10)	18.85 (0.84)	1.01 (0.22)	209.39 (8.84)
*Females*								
<125%	820	4.22 (0.76)	192.32 (34.73)	8.91 (2.23)	0.47 (0.06)	17.45 (0.55)	0.56 (0.18)	223.92 (34.07)
>125%	1048	8.61 (0.50)	199.46 (23.94)	12.74 (1.44)	0.65 (0.06)	16.31 (0.87)	1.04 (0.25)	238.81 (23.99)
**Race**								
*African American*								
<125%	982	3.48 (0.81)	116.23 (13.57)	12.41 (2.55)	0.39 (0.03)	15.24 (0.78)	0.44 (0.12)	148.18 (11.30)
>125%	1047	4.63 (0.31)	138.61 (10.73)	17.96 (2.45)	0.53 (0.04)	15.52 (1.06)	0.65 (0.13)	177.89 (11.12)
*White*								
<125%	439	4.11 (1.83)	377.28 (104.05)	8.47 (2.06)	0.68 (0.10)	24.73 (2.19)	0.81 (0.37)	416.09 (104.05)
>125%	950	13.06 (1.63)	265.96 (64.53)	10.62 (1.24)	0.91 (0.11)	21.31 (2.10)	1.73 (0.63)	313.59 (63.41)
**Age, years**								
*30–49*								
≤125%	812	4.79 (1.69)	215.90 (56.04)	9.90 (2.09)	0.51 (0.05)	18.95 (1.69)	0.58 (0.14)	250.62 (56.00)
>125%	1075	7.12 (1.15)	190.00 (13.27)	14.66 (1.91)	0.70 (0.07)	18.03 (0.61)	0.97 (0.18)	231.48 (13.07)
*50–64*								
≤125%	609	4.28 (0.98)	158.40 (20.76)	11.83 (2.21)	0.48 (0.07)	16.85 (0.62)	0.73 (0.34)	192.58 (19.27)
>125%	922	8.38 (1.29)	171.84 (15.60)	16.45 (3.30)	0.62 (0.04)	16.80 (0.96)	1.12 (0.43)	215.21 (15.25)

Abbreviations: HANDLS, Healthy Aging in Neighborhoods of Diversity across the Life Span. ^1^ To permit extrapolation to the population of interest, sample weights were applied to all estimates and the sample design was accounted for in the calculation of standard errors. ^2^ Estimates adjusted for energy, sex (as appropriate), race (as appropriate), and age. Although untransformed estimates are presented, significance results are based on transformed estimates [51]. ^3^ Health and Human Services 2004 poverty guidelines, income expressed as a percentage of poverty [42].

**Table 6:** Mean flavonoid intakes ^1,2^ (mg), for all and by race, HANDLS study and WWEIA, NHANES 2007–2010, 1 day.

Flavonoid Class	All		African American/Black ^3^		White	
	HANDLS	WWEIA, NHANES ^4^	HANDLS	WWEIA, NHANES ^4^	HANDLS	WWEIA, NHANES ^4^
*n*	3418	2598	2029	895	1389	1703
	------------------------------------------------------------*milligrams (SE)*------------------------------------------------------------------					
Anthocyanidins	10.19 * (1.24)	12.08 (1.77)	4.34 * (0.34)	6.44 (0.85)	12.59 * (1.82)	13.41 (2.16)
Flavan-3-ols	243.81(24.81)	241.47 (19.29)	127.46(9.86)	144.09 (12.72)	288.18* 69.69)	264.39 (23.23)
Flavanones	12.36 (1.96)	10.95 (1.04)	16.67 (2.31)	14.54 (1.58)	10.41 (1.11)	10.11 (1.15)
Flavones	0.79 * (0.05)	0.84 (0.06)	0.48 * (0.05)	0.54 (0.02)	0.88 (0.11)	0.91 (0.07)
Flavonols	21.17 (0.80)	22.06 (0.92)	15.38 (0.81)	16.05 (0.52)	22.20 (2.09)	23.47 (1.13)
Isoflavones	1.03 (0.19)	1.09 (0.16)	0.53 * (0.11)	0.83 (0.16)	1.55 * (0.59)	1.15 (0.18)
Total	289.34 (24.56)	288.49 (20.33)	164.86 (9.13)	182.50 (13.47)	335.81* (68.78)	313.44 (24.56)

Abbreviations: HANDLS, Healthy Aging in Neighborhoods of Diversity across the Life Span, WWEIA-NHANES, What We Eat in America-National Health and Nutrition Examination Survey. * Within flavonoid class, intake estimate is significantly different from that of individuals in NHANES (*p* < 0.01). ^1^ To permit extrapolation to the population of interest, sample weights from the respective surveys were applied to all estimates and the sample designs were accounted for in the calculation of standard errors. ^2^ All estimates adjusted for energy, race (as appropriate), income, and age. Although untransformed estimates are presented, significance results are based on transformed estimates [51]. ^3^ Race labeled as African American in HANDLS and black in NHANES. ^4^ WWEIA, NHANES sample restricted to non-pregnant, non-lactating individuals (black and white, or the designated race) who are age 30–64 with a household income <$75,000.

## References

[1] Healthy People 2020. https://www.healthypeople.gov/.

[2] Wirth M.D., Hébert J.R., Shivappa N., Hand G.A., Hurley T.G., Drenowatz C., McMahon D., Shook R.P., Blair S.N. (2016). Anti-inflammatory dietary inflammatory index scores are associated with healthier scores on other dietary indices. Nutr. Res..

[3] Ahluwalia N., Andreeva V.A., Kesse-Guyot E., Hercberg S. (2012). Dietary patterns, inflammation and the metabolic syndrome. Diabetes Metab..

[4] Hotamisligil G.S. (2006). Inflammation and metabolic disorders. Nature.

[5] Gregor M.F., Hotamisligil G.S. (2011). Inflammatory mechanisms in obesity. Annu. Rev. Immunol..

[6] Alkhalidy H., Wang Y., Liu D. (2018). Dietary flavonoids in the prevention of T2D: An overview. Nutrients.

[7] Cassidy A. (2018). Berry anthocyanin intake and cardiovascular health. Mol. Aspects Med..

[8] Eggersdorfer M., Wyss A. (2018). Carotenoids in human nutrition and health. Arch. Biochem. Biophys..

[9] Williamson G. (2017). The role of polyphenols in modern nutrition. Nutr. Bull..

[10] Scalbert A., Williamson G. (2000). Dietary intake and bioavailability of polyphenols. J. Nutr..

[11] Beecher G.R. (2003). Overview of dietary flavonoids: Nomenclature, occurrence and intake. J. Nutr..

[12] Basu P., Maier C. (2016). In vitro antioxidant activities and polyphenol contents of seven commercially available fruits. Pharmacogn. Res..

[13] Costa C., Tsatsakis A., Mamoulakis C., Teodoro M., Briguglio G., Caruso E., Tsoukalas D., Margina D., Dardiotis E., Kouretas D. (2017). Current evidence on the effect of dietary polyphenols intake on chronic diseases. Food Chem. Toxicol..

[14] Panche A.N., Diwan A.D., Chandra S.R. (2016). Flavonoids: An overview. J. Nutr. Sci..

[15] Kim Y., Je Y. (2017). Flavonoid intake and mortality from cardiovascular disease and all causes: A meta-analysis of prospective cohort studies. Clin. Nutr. ESPEN.

[16] Kim K., Vance T.M., Chun O.K. (2016). Greater flavonoid intake is associated with improved CVD risk factors in US adults. Br. J. Nutr..

[17] Dower J.I., Geleijnse J.M., Gijsbers L., Zock P.L., Kromhout D., Hollman P.C.H. (2015). Effects of the pure flavonoids epicatechin and quercetin on vascular function and cardiometabolic health: A randomized double-blind, placebo-controlled, crossover trial. Am. J. Clin. Nutr..

[18] Yamagata K., Tagami M., Yamori Y. (2015). Dietary polyphenols regulate endothelial function and prevent cardiovascular disease. Nutrition.

[19] Cassidy A., Huang T., Rice M.S., Rimm E.B., Tworoger S.S. (2014). Intake of dietary flavonoids and risk of epithelial ovarian cancer. Am. J. Clin. Nutr..

[20] Woo H.D., Lee J., Choi I.J., Kim C.G., Lee J.L., Kwon O., Kim J. (2014). Dietary flavonoids and gastric cancer risk in a korean population. Nutrients.

[21] Wang G., Wang J., Du L., Li F. (2015). Effect and mechanism of total flavonoids extracted from cotinus coggygria against glioblastoma cancer in vitro and in vivo. BioMed Res. Int..

[22] Fantini M., Benvenuto M., Masuelli L., Frajese G.V., Tresoldi I., Modesti A., Bei R. (2015). In vitro and in vivo antitumoral effects of combinations of polyphenols, or polyphenols and anticancer drugs: Perspectives on cancer treatment. Int. J. Mol. Sci..

[23] Ganai A.A., Farooqi H. (2015). Bioactivity of genistein: A review of in vitro and in vivo studies. Biomed. Pharmacother..

[24] Zamora-Ros R., Forouhi N.G., Sharp S.J., González C.A., Buijsse B., Guevara M., van der Schouw Y.T., Amiano P., Boeing H., Bredsdorff L. (2014). Dietary intakes of individual flavanols and flavonols are inversely associated with incident type 2 diabetes in european populations. J. Nutr..

[25] Gharib A., Faezizadeh Z., Godarzee M. (2013). Treatment of diabetes in the mouse model by delphinidin and cyanidin hydrochloride in free and liposomal forms. Planta Med..

[26] Bao T., Wang Y., Li Y., Gowd V., Niu X., Yang H., Chen L., Chen W., Sun C. (2016). Antioxidant and antidiabetic properties of tartary buckwheat rice flavonoids after in vitro digestion. J. Zhejiang Univ. Sci. B.

[27] Testa R., Bonfigli A.R., Genovese S., De Nigris V., Ceriello A. (2016). The possible role of flavonoids in the prevention of diabetic complications. Nutrients.

[28] Gil-Cardoso K., Ginés I., Pinent M., Ardévol A., Blay M., Terra X. (2016). Effects of flavonoids on intestinal inflammation, barrier integrity and changes in gut microbiota during diet-induced obesity. Nutr. Res. Rev..

[29] Huang J., Wang Y., Xie Z., Zhou Y., Zhang Y., Wan X. (2014). The anti-obesity effects of green tea in human intervention and basic molecular studies. Eur. J. Clin. Nutr..

[30] Guo H., Ling W. (2015). The update of anthocyanins on obesity and type 2 diabetes: Experimental evidence and clinical perspectives. Rev. Endocr. Metab. Disord..

[31] Cassidy A., Rimm E.B., O’Reilly E.J., Logroscino G., Kay C., Chiuve S.E., Rexrode K.M. (2012). Dietary flavonoids and risk of stroke in women. Stroke.

[32] Cassidy A., Bertoia M., Chiuve S., Flint A., Forman J., Rimm E.B. (2016). Habitual intake of anthocyanins and flavanones and risk of cardiovascular disease in men. Am. J. Clin. Nutr..

[33] Bao Y., Bertoia M.L., Lenart E.B., Stampfer M.J., Willett W.C., Speizer F.E., Chavarro J.E. (2016). Origin, methods, and evolution of the three nurses’ health studies. Am. J. Public Health.

[34] Harvard T.H. Chan School of Public Health. Health Professionals Follow-Up Study. https://sites.sph.harvard.edu/hpfs/about-the-study/.

[35] Goetz M.E., Judd S.E., Safford M.M., Hartman T.J., McClellan W.M., Vaccarino V. (2016). Dietary flavonoid intake and incident coronary heart disease: The REasons for geographic and racial differences in stroke (REGARDS) study. Am. J. Clin. Nutr..

[36] Goetz M.E., Judd S.E., Hartman T.J., McClellan W., Anderson A., Vaccarino V. (2016). Flavanone intake is inversely associated with risk of incident ischemic stroke in the REasons for geographic and racial differences in stroke (REGARDS) study. J. Nutr..

[37] (1996). NHLBI Stroke Belt Initiative. https://www.nhlbi.nih.gov/files/docs/resources/heart/sb_spec.pdf.

[38] Evans M.K., Lepkowski J.M., Powe N.R., LaVeist T., Fanelli Kuczmarski M., Zonderman A.B. (2010). Healthy aging in neighborhoods of diversity across the life span (HANDLS): Overcoming barriers to implementing a longitudinal, epidemiologic, urban study of health, race, and socioeconomic status. Ethn. Dis..

[39] Fanelli Kuczmarski M., Bodt B.A., Stave Shupe E., Zonderman A.B., Evans M.K. (2018). Dietary patterns associated with lower 10-year atherosclerotic cardiovascular disease risk among urban African-American and white adults consuming western diets. Nutrients.

[40] Statovci D., Aguilera M., MacSharry J., Melgar S. (2017). The impact of western diet and nutrients on the microbiota and immune response at mucosal interfaces. Front. Immunol..

[41] Sebastian R.S., Wilkinson E.C., Goldman J.D., Martin C.L., Steinfeldt L.C., Murayi T., Moshfegh A.J. (2015). A new database facilitates characterization of flavonoid intake, sources, and positive associations with diet quality among US adults. J. Nutr..

[42] Office of the Assistant Secretary for Planning and Evaluation 2004 HHS Poverty Guidelines. https://aspe.hhs.gov/2004-hhs-poverty-guidelines.

[43] Healthy Aging in Neighborhoods of Diversity across the Life Span. Study Protocols. https://handls.nih.gov/02Protocol.htm.

[44] Ware J.E., Kosinski M., Keller S.D. (1998). SF-12: How to Score the SF-12 Physical and Mental Health Summary Scales.

[45] Wilkinson G. (1993). The Wide Range Achievement Test—3rd Edition (WRAT-3). https://www.unc.edu/depts/sph/longscan/pages/measures/Ages12to14/writeups/Age%2012%20WRAT-3.pdf.

[46] Moshfegh A.J., Rhodes D.G., Baer D.J., Murayi T., Clemens J.C., Rumpler W.V., Paul D.R., Sebastian R.S., Kuczynski K.J., Ingwersen L.A. (2008). The U.S. Department of Agriculture automated multiple-pass method reduces bias in the collection of energy intakes. Am. J. Clin. Nutr..

[47] Raper N., Perloff B., Ingwersen L., Steinfeldt L., Anand J. (2004). An overview of USDA’s Dietary Intake Data System. J. Food Compost. Anal..

[48] Food Surveys Research Group: Beltsville, MD United States Department of Agriculture. https://www.ars.usda.gov/northeast-area/beltsville-md-bhnrc/beltsville-human-nutrition-research-center/food-surveys-research-group/.

[49] What We Eat in America Food Categories. United States Department of Agriculture Web Site. https://www.ars.usda.gov/ARSUserFiles/80400530/pdf/1314/food_category_list.pdf.

[50] Flavonoid Database Documentation. https://www.ars.usda.gov/ARSUserFiles/80400530/pdf/fndds/FlavonoidDB_documentation_0710.pdf.

[51] Albert J. (2009). Bayesian Computation with R.

[52] Bai W., Wang C., Ren C. (2013). Intakes of total and individual flavonoids by US adults. Int. J. Food Sci. Nutr..

[53] Fanelli Kuczmarski M., Sees A.C., Hotchkiss L., Cotugna N., Evans M.K., Zonderman A.B. (2010). Higher healthy eating index-2005 scores associated with reduced symptoms of depression in an urban population: Findings from the healthy aging in neighborhoods of diversity across the life span (HANDLS) study. J. Am. Diet. Assoc..

[54] Fanelli Kuczmarski M., Beydoun M.A., Cotugna N., Daniels L., Mason M.A., Zonderman A.B., Evans M.K. (2016). Literacy contributes to greater higher diet quality in a socioeconomically diverse urban prospective cohort. Top. Clin. Nutr..

[55] Darmon N., Drewnowski A. (2015). Contribution of food prices and diet cost to socioeconomic disparities in diet quality and health: A systematic review and analysis. Nutr. Rev..

[56] Chun O.K., Lee S.G., Wang Y., Vance T., Song W.O. (2012). Estimated flavonoid intake of the elderly in the united states and around the world. J. Nutr. Gerontol. Geriatr..

[57] Drewnowski A., Kawachi I. (2015). Diets and health: How food decisions are shaped by biology, economics, geography, and social interactions. Big Data.

[58] Sak K. (2017). Current epidemiological knowledge about the role of flavonoids in prostate carcinogenesis. Exp. Oncol..

[59] Beydoun M.A., Fanelli-Kuczmarski M., Allen A., Beydoun H.A., Popkin B.M., Evans M.K., Zonderman A.B. (2015). Monetary value of diet is associated with dietary quality and nutrient adequacy among urban adults, differentially by sex, race and poverty status. PLoS ONE.

[60] Allcott H., Diamond R., Dube J.-P. (2017). The Geography of Poverty and Nutrition: Food Deserts and Food Choices across the United States.

[61] Casagrande S.S., Franco M., Gittelsohn J., Zonderman A.B., Evans M.K., Kuczmarski M.F., Gary-Webb T. (2011). Neighborhood characteristics and availability of healthy foods in Baltimore. Public Health Nutr..

[62] Vaughan C.A., Collins R., Ghosh-Dastidar M., Beckman R., Dubowitz T. (2017). Does where you shop or who you are predict what you eat? The role of stores and individual characteristics in dietary intake. Prev. Med..

[63] Current Population Survey Data for Social, Economic, and Health Research. IPUMS-CPS Web Site. https://www.census.gov/programs-surveys/cps.html.

[64] U.S. Department of Labor, Bureau of Labor Statistics from the Current Population Survey, 2005 Annual Averages. https://www.bls.gov/cps/aa2005/cpsaat3.pdf.

[65] Milicic S., DeCicca P. (2017). The impact of economic conditions on healthy dietary intake: Evidence from fluctuations in Canadian unemployment rates. J. Nutr. Educ. Behav..

[66] Fanelli Kuczmarski M., Mason M.A., Beydoun M.A., Allegro D., Zonderman A.B., Evans M.K. (2013). Dietary patterns and sarcopenia in an urban African American and White population in the United States. J. Nutr. Gerontol. Geriatr..

[67] Fanelli Kuczmarski M., Mason M., Allegro D., Beydoun M., Zonderman A.B., Evans M.K. (2015). Dietary quality and nutritional biomarkers associated with dietary patterns of socioeconomically diverse urban African American and White population. Procedia Food Sci..

[68] Rhodes D.G., Murayi T., Clemens J.C., Baer D.J., Sebastian R.S., Moshfegh A.J. (2013). The USDA Automated Multiple-Pass Method accurately assesses population sodium intakes. Am. J. Clin. Nutr..

[69] Naska A., Lagiou A., Lagiou P. (2017). Dietary assessment methods in epidemiological research: Current state of the art and future prospects. F1000Research.

[70] Agudo A. (2004). Measuring Intake of Fruit and Vegetable Intake.

